# Dietary Soy Supplement on Fibromyalgia Symptoms: A Randomized, Double-Blind, Placebo-Controlled, Early Phase Trial

**DOI:** 10.1093/ecam/nen069

**Published:** 2011-06-23

**Authors:** Dietlind L. Wahner-Roedler, Jeffrey M. Thompson, Connie A. Luedtke, Susan M. King, Stephen S. Cha, Peter L. Elkin, Barbara K. Bruce, Cynthia O. Townsend, Jody R. Bergeson, Andrea L. Eickhoff, Laura L. Loehrer, Amit Sood, Brent A. Bauer

**Affiliations:** ^1^Division of General Internal Medicine, Mayo Clinic, Rochester, MN, USA; ^2^The Fibromyalgia Treatment Program, Mayo Clinic, Rochester, MN, USA; ^3^The Fibromyalgia Treatment/Rehabilitation Center, Mayo Clinic, Rochester, MN, USA; ^4^The Division of Biostatistics, Mayo Clinic, Rochester, MN, USA; ^5^The Division of Tertiary Psychiatry and Psychology, Mayo Clinic, Rochester, MN, USA

## Abstract

Most patients with fibromyalgia use complementary and alternative medicine (CAM). Properly designed controlled trials are necessary to assess the effectiveness of these practices. This study was a randomized, double-blind, placebo-controlled, early phase trial. Fifty patients seen at a fibromyalgia outpatient treatment program were randomly assigned to a daily soy or placebo (casein) shake. Outcome measures were scores of the Fibromyalgia Impact Questionnaire (FIQ) and the Center for Epidemiologic Studies Depression Scale (CES-D) at baseline and after 6 weeks of intervention. Analysis was with standard statistics based on the null hypothesis, and separation test for early phase CAM comparative trials. Twenty-eight patients completed the study. Use of standard statistics with intent-to-treat analysis showed that total FIQ scores decreased by 14% in the soy group (*P* = .02) and by 18% in the placebo group (*P* < .001). The difference in change in scores between the groups was not significant (*P* = .16). With the same analysis, CES-D scores decreased in the soy group by 16% (*P* = .004) and in the placebo group by 15% (*P* = .05). The change in scores was similar in the groups (*P* = .83). Results of statistical analysis using the separation test and intent-to-treat analysis revealed no benefit of soy compared with placebo. Shakes that contain soy and shakes that contain casein, when combined with a multidisciplinary fibromyalgia treatment program, provide a decrease in fibromyalgia symptoms. Separation between the effects of soy and casein (control) shakes did not favor the intervention. Therefore, large-sample studies using soy for patients with fibromyalgia are probably not indicated.

## 1. Introduction

Fibromyalgia syndrome (FMS) is a chronic, generalized pain syndrome that affects the musculoskeletal system [1]. This syndrome is typically diagnosed in patients who experience generalized musculoskeletal pain and have excessive tenderness in at least 11 of 18 specific points [2]. Although the primary cause of FMS is unclear, a growing body of evidence indicates that the widespread pain associated with this syndrome is due to abnormalities in the central nervous system. Therefore, drug therapy for FMS is most often aimed at the central nervous system and includes tricyclic antidepressants, selective serotonin reuptake inhibitors, dual serotonin and norepinephrine reuptake inhibitors, analgesics and anticonvulsants [1].

In addition to medical therapies, complementary and alternative medicine (CAM) therapies have been used to treat FMS symptoms [3]. Overall, >50% of patients with rheumatologic conditions, including FMS, use CAM therapies [4–6]. Wahner-Roedler et al. [6] reported that 98% of patients who are referred to a fibromyalgia treatment program may have used some form of CAM within the last 6 months. The types of CAM therapies used to reduce FMS symptoms include massage, meditation, acupuncture, hypnotherapy and dietary supplementation. Few CAM interventions have been adequately tested in controlled clinical trials. In a systematic review published in 2003, Holdcraft et al. [3] concluded that the strongest evidence for efficacy in FMS exists for acupuncture. Magnesium use, *S*-adenosyl-L-methionine (SAMe) use, and massage therapy have moderate evidence; chlorella use, relaxation, biofeedback, magnet therapies, homeopathy, botanical oils, balneotherapy and use of anthocyanidins have limited evidence. In a 2004 published summary of CAM trial data, Ernst [7] concluded that acupuncture and spinal manipulations have shown a significant promise in the treatment of FMS. No dietary supplement has conclusive evidence of efficacy in the treatment of FMS symptoms.

Soy is a widely used dietary supplement that has not been previously tested for treating FMS. On the basis of studies indicating that dietary soy relieves neuropathic pain in animals [8–11] and reduces pain and improves range of motion of the knee joints in humans with osteoarthritis [12], we hypothesized that soy consumption might improve FMS symptoms. The aim of our study was to evaluate whether dietary soy supplement can improve symptoms in patients with FMS participating in a 1.5-day multidisciplinary fibromyalgia treatment program, as measured by the Fibromyalgia Impact Questionnaire (FIQ) and the Center for Epidemiologic Studies Depression Scale (CES-D).

## 2. Subjects and Methods

This study was approved by the Mayo Clinic Institutional Review Board and registered as NCT00279942 in Clinical Trials.gov.

**Figure 1:**
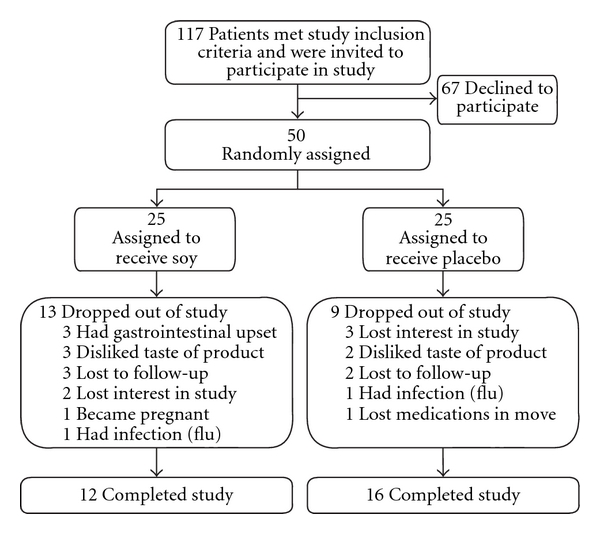
Flow chart of patients in the placebo-controlled soy supplement trial.

Patients presenting to the Mayo Fibromyalgia Treatment Program between May 2006 and August 2006 were invited to participate in this trial. Exclusion criteria included diagnoses of bipolar disorder, schizophrenia, dementia, diabetes mellitus and inflammatory bowel disease; allergy to soy or other study product ingredients; pregnancy; and consumption of soy products within the past 30 days. Among patients seen in our Fibromyalgia Treatment Program between May 2006 and August 2006, 117 met the study inclusion criteria and were invited to participate in the study (Figure 1). Of these, 67 patients declined to participate, and the remaining 50 patients were randomly assigned to either soy supplement or placebo.

### 2.1. The Mayo Fibromyalgia Treatment Program

The Mayo Fibromyalgia Treatment Program is a 1.5 day, multidisciplinary outpatient program staffed by physicians from the Department of Physical Medicine and Rehabilitation and the Department of Rheumatology. Access to this program is limited to patients with a presumed diagnosis of FMS who are referred by Mayo Clinic physicians. The patients undergo an initial evaluation by a registered nurse specifically trained in rheumatologic disorders, with the collaboration of a physician to confirm the diagnosis. Only patients with a confirmed diagnosis of FMS as defined by the American College of Rheumatology in 1990—namely, widespread musculoskeletal pain of at least 3 months' duration and excessive tenderness in at least 11 of 18 predefined anatomical sites [2]—are enrolled in the program. Components of the program are carried out by a core group of team members that includes registered nurses, rheumatologists, psychiatrists, occupational therapists, physical therapists and ancillary staff. The mission of the fibromyalgia treatment program is to improve patients' physical and mental health functioning, impart evidence-based information and create a standardized treatment approach. Further details about this multidisciplinary program have been described [13].

### 2.2. Study Design

We conducted a randomized, double-blind, placebo-controlled, early phase trial. Participants were randomly assigned to either soy supplement or placebo when they started the fibromyalgia treatment program. An informed consent was obtained from all participants. The participants completed the FIQ and the CES-D at baseline and were randomly assigned to either soy supplement or placebo taken once a day for 6 weeks. They were asked to collect wrappers of the product and to send them by mail in a provided envelope addressed to Mayo Clinic, together with another set of the completed FIQ and CES-D forms, at the end of the 6 weeks. The study coordinator, for whom the assignment of patients was blinded, called the participants weekly to inquire about product tolerance and compliance.

### 2.3. Products

The treatment was a soy shake (provided by Physicians Pharmaceuticals, Inc, Kernersville, NC, USA) that contained 20 g of soy protein and 160 mg of soy isoflavone. The placebo was a shake that contained 20 g of milk-based protein (casein) and no isoflavone (provided by Physicians Pharmaceuticals, Inc). Flavoring, sweetening and nutritional content were identical in the two shakes.

### 2.4. Instruments

#### 2.4.1. Fibromyalgia Impact Questionnaire

The FIQ is a validated questionnaire that was developed to measure status, progress and outcomes of people with FMS [14]. A self-administered instrument, it takes *∼*5 min to complete. The FIQ contains 20 items that measure physical functioning; symptoms of pain, fatigue, morning tiredness and stiffness; job difficulty; depression and anxiety; days of work missed; and overall well-being of the person during the previous week. A higher score indicates a greater effect of FMS on the person, with a range of total score from 0 to 100.

Questions 1 through 11 rate the ability to complete various activities and are scored and summed to yield 1 physical impairment score (0, no impairment; 10, maximum impairment). Question 12 inquires about the number of days out of the past 7 when the patient felt good, and question 13 inquires about the number of days during the past week when the patient missed work, including housework, because of fibromyalgia, with each question yielding a separate score (0, no impairment; 10, maximum impairment). Questions 14 through 20 are a series of visual analog scales (range, 0–10) for rating the various symptoms characteristic of FMS (0, no impairment; 70, maximum impairment).

#### 2.4.2. Center for Epidemiologic Studies Depression Scale

The CES-D is a 20-item measure of depression. Its questions represent depressed mood; feelings of guilt, worthlessness, helplessness or hopelessness; psychomotor retardation; loss of appetite; and sleep disturbance [15]. Scale scores can range from 0 to 60; a higher number indicates greater depression. Various cutoff points for depression have been used. For example, Weissman et al. [16] used the cutoff of 16 and Turk et al. [17] the cutoff of 19.

### 2.5. Statistical Analysis

Patient demographics were summarized using descriptive statistics. The mean (±SD) of the total FIQ scores and the CES-D scores at baseline and at 6 weeks between the soy group and the placebo group were analyzed by Wilcoxon rank sum test. The difference and relative change (%) of the FIQ and CES-D scores at 6 weeks from baseline were also compared by Wilcoxon rank sum test. The comparison of the difference from baseline to 6 weeks within the soy and placebo groups was analyzed by the Wilcoxon signed rank test. The analyses were performed using intent-to-treat and per-protocol approaches. In intent-to-treat analysis, a participant who did not complete the entire 6-week supplement trial or who failed to complete the FIQ and CES-D forms was considered a dropout.

Because this study was an early phase trial with a small sample size, we also analyzed our data by using the separation test, as described by Aickin [18, 19], to assess whether it is worthwhile to pursue research on soy supplementation for patients with fibromyalgia. By use of this test, the standard deviation of the effect estimate (SDE) of the mean difference can be found. The value of Δ = 1.645* SDE is then calculated. If the mean difference exceeds Δ/2 (in the favorable direction), further research is recommended; if it decreases below −Δ/2 (in the unfavorable direction), further research is not recommended. Otherwise, the statement is made that there is not enough information to make a recommendation.

## 3. Results

### 3.1. Baseline Characteristics

A total of 50 patients (49 women) were recruited for this trial. Median age was 47.7 years (range, 18–76 years). There was no significant difference in age (*P* = .99), FIQ scores (*P* = .36) or CES-D scores (*P* = .48) between the two groups. Twenty-eight patients (56%)—12 in the soy group and 16 in the placebo group (*P* = .39)—completed the 6-week trial. Reasons for not completing it are depicted in Figure 1. Patients who did not finish the trial were significantly younger (median age, 39.8 years) than those who finished it (median age, 53.9 years) (*P* < .001). The median FIQ score was higher for patients who did not complete the trial (59.5) than for those who completed the study (54.8), but the difference was not statistically significant (*P* = .12). The median CES-D score also was higher for patients who did not complete the trial (26.5) than for patients who completed the trial (14.0), but this also was not a statistically significant difference (*P* = .14).

### 3.2. Between-Group Comparisons

**Figure 2:**
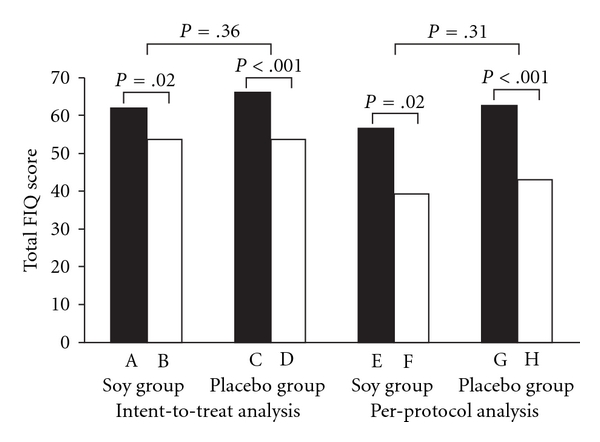
Total FIQ scores of patients in soy supplement trial, with intent-to-treat analysis (A–D) and per–protocol analysis (E–H). Score of patients randomly assigned to receive soy at study entry (A) and score of patients after 6 weeks of soy treatment (B). Score of patients randomly assigned to receive placebo at study entry (C) and score of patients after 6 weeks of placebo (D). Score of patients randomly assigned to receive soy at study entry (E) and score of patients after 6 weeks of soy treatment (F). Score of patients randomly assigned to receive placebo at study entry (G) and score of patients after 6 weeks of placebo (H).

**Figure 3:**
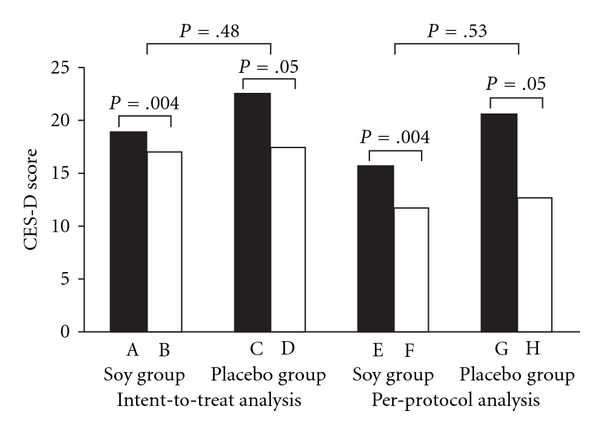
CES-D scores of patients in soy supplement trial, with intent-to-treat analysis (A–D) and per-protocol analysis (E–H). Score of patients randomly assigned to receive soy at study entry (A) and score of patients after 6 weeks of soy treatment (B). Score of patients randomly assigned to receive placebo at study entry (C) and score of patients after 6 weeks of placebo (D). Score of patients randomly assigned to receive soy at study entry (E) and score of patients after 6 weeks of soy treatment (F). Score of patients randomly assigned to receive placebo at study entry (G) and score of patients after 6 weeks of placebo (H).

With intent-to-treat analysis, the total FIQ scores determined at study entry and at study completion decreased 14% (±29) in the soy group and 18% (±25) in the placebo group (*P* = .16). With per-protocol analysis, the total FIQ scores decreased 29% (±36) in the soy group and 28% (±26) in the placebo group (*P* = .93) (Figure 2). No statistically significant decrease between the soy group and the placebo group was observed for any of the FIQ subclass scores. With intent-to-treat analysis, the CES-D scores improved 16% (±26) in the soy group and 15% (±41) in the placebo group (*P* = .83); with per-protocol analysis, the CES-D scores improved 33% (±30) in the soy group and 24% (±50) in the placebo group (Figure 3). However, the decrease between the groups was not statistically significant (*P* = .96). Using various cutoff points for depression (CES-D ≥ 16, CES-D ≥ 19 and CES-D ≥ 27) and intent-to-treat analysis, we found a depression rate of 52%, 48% and 28%, respectively, in the soy group and 52%, 48% and 32% in the control group at study entry. After 6 weeks, these percentages were 48%, 44% and 24% in the treatment group and 48%, 36% and 24% in the control group. There was no statistically significant difference between the groups (*P* = 1.00). Determined by using per-protocol analysis for these three different CES-D scores, the percentages of patients with depression at study entry in the treatment group were 42%, 33% and 8% and in the control group were 38%, 38% and 19%. After 6 weeks, 33% of the treatment group had CES-D scores of 16 or greater, 25% had scores of 19 or greater and 0% had scores of 27 or greater. The percentages for the control group were 31%, 19% and 6%, respectively. There was no statistically significant difference between the groups.

The separation test using the difference from baseline to 6 weeks showed no benefit for soy, as determined by FIQ scores and CES-D scores for both intent-to-treat and per-protocol analyses. We did not have enough information to make any recommendation for or against the use of soy using the separation test per-protocol analysis based on total FIQ scores (Table 1).

### 3.3. Within-Group Comparisons

Significant, but modest, improvement in total FIQ scores (soy group, *P* = .02; placebo group, *P* < .001) (Figure 2) and CES-D scores (soy group, *P* = .004; placebo group, *P* = .05) (Figure 3) between study entry and study completion was seen in both groups. Using three different cutoff points for depression (CES-D ≥ 16, CES-D ≥ 19 and CES-D ≥ 27), we found no significant improvement using the McNemar test for the soy and control groups (*P* > .50).

#### 3.3.1. FIQ Subclass Scores

The average score of answers to questions 1 through 11 showed no significant improvement from before treatment to after treatment in both groups, as did the average score of answers to questions 12 and 13. However, the average score of answers to questions 14 through 20 showed significant improvement from before treatment to after treatment in the soy group (*P* = .004) and the placebo group (*P* = .001).

## 4. Discussion

The present study shows that use of soy product and use of the chosen placebo for 6 weeks, when combined with an educational intervention, were both associated with modest improvement in symptoms of fibromyalgia and depression.

Patients seen in the Mayo Fibromyalgia Treatment Program have moderate to severe symptoms, as demonstrated by the high total FIQ score of our sample at study entry (average score, 59.4 ± 13.3). A 1.5-day multidisciplinary treatment program, such as the program developed at our institution is considered standard care for these patients and has been shown to have a significant positive effect on the impact of illness among patients with FMS [13]. Despite participation in such multidisciplinary treatment programs and use of many conventional medications (e.g., tricyclic antidepressants, selective serotonin reuptake inhibitors, dual serotonin and norepinephrine reuptake inhibitors, analgesics, anticonvulsants), most patients with FMS continue to have functionally limiting symptoms [20].

Soy consumption has been reported in the CAM literature to have many beneficial effects on bone health, the cardiovascular system and degenerative arthritis and has been shown in epidemiologic studies to be associated with lower risk of several cancers [21]. Intrigued by animal studies indicating that dietary soy provided relief in neuropathic pain, we decided to perform this early phase trial [8–11]. Using standard statistics based on the null hypothesis, we show that the use of dietary soy is no more helpful than the use of casein when each is combined with a brief multidisciplinary treatment program. Although we cannot exclude the high drop-out rate, a patient population with severe symptoms as a result of referral bias, and a lack of efficacy of the soy product as reasons for these findings, another possible cause is the small sample size of our study, which represents an early phase trial. In concordance with Aickin [18, 19], we believe that early phase research in CAM is important because of its large influence on the subsequent expenditure of money and effort in CAM therapies. By analyzing our data with separation testing, we were unable to document any separation between soy use and casein use (control) through FIQ and CES-D scores in favor of soy when using intent-to-treat analysis, as well as per-protocol analysis. Our interpretation of these results is that further studies using large sample sizes of patients with fibromyalgia are unlikely to show a positive effect of soy supplement compared with placebo and therefore are not indicated.

Given the prevalence of CAM, the many various CAM therapies used for patients with FMS, and the cost associated with their use, it appears prudent that each of these therapies should be carefully assessed, initially with early phase trials. If the early phase trials result in promising findings, adequately powered clinical trials should be performed.

The results of this randomized, double-blind, placebo-controlled, early phase trial of soy shakes for patients with FMS suggests that, on the basis of FIQ scores and CES-D scores, dietary soy supplementation is no more beneficial than casein shakes.

## Figures and Tables

**Table 1:** Summary of data analysis with a separation test ^a^.

Outcome	Control (placebo)		Treatment (soy)		SDE	Δ/2 ^b^	Mean difference (placebo/soy)	Separation in favor of placebo or soy ^c^	Further research with soy indicated?
	Mean change in scores	95% CI	Mean change in scores	95% CI					

Intent-to-treat analysis									
F1-11^d^	−0.51	−3.55 to 2.54	−0.07	−1.93 to 1.79	0.18	0.15	−0.44	Placebo	No
F12^d^	−2.06	−8.69 to 4.57	−1.26	−7.00 to 4.48	0.45	0.37	−0.80	Placebo	No
F13^d^	−1.12	−7.01 to 4.77	−0.32	−5.08 to 4.44	0.39	0.32	−0.80	Placebo	No
F14-20^d^	−8.84	−38.73 to 21.05	−6.56	−29.36 to 16.24	1.91	1.57	−2.28	Placebo	No
Total FIQ scores	−12.52	−53.34 to 28.29	−8.21	−39.39 to 22.97	2.61	2.15	−4.32	Placebo	No
Total CES-D scores	−5.12	−31.60 to 21.36	−1.92	−8.12 to 4.28	1.39	1.15	−3.20	Placebo	No

Per-protocol analysis									
F1-11^d^	−0.79	−4.52 to 2.94	−0.15	−2.89 to 2.59	0.32	0.27	−0.64	Placebo	No
F12^d^	−3.22	−10.63 to 4.20	−2.62	−10.16 to 4.92	0.71	0.58	−0.60	Placebo	No
F13^d^	−1.75	−8.89 to 5.39	−0.67	−7.63 to 6.29	0.68	0.56	−1.08	Placebo	No
F14-20^d^	−13.81	−47.69 to 20.07	−13.67	−40.64 to 13.30	2.95	2.42	−0.15	Neither	…^e^
Total FIQ scores	−19.57	−65.40 to 26.26	−17.10	−55.62 to 21.42	4.06	3.34	−2.47	Neither	…
Total CES-D scores	−8.00	−40.05 to 24.05	−4.00	−10.99 to 2.99	2.37	1.95	−4.00	Placebo	No

^a^As described by Aickin [18, 19];  ^b^Δ/2 = 1.645∗SDE/2;  ^c^If the mean difference exceeds Δ/2 (in the favorable direction, positive for soy), further research is recommended;  ^d^F1-11, questions 1-11 of FIQ, which rate the ability to complete various activities and are scored and summed to yield 1 physical impairment score (0, no impairment; 10, maximum impairment); F12, question 12 of FIQ, which inquires about the number of days out of the past 7 days when the patient felt well (0, no impairment; 10, maximum impairment); F13, question 13 of FIQ, which inquires about the number of days during the past week when the patient missed work, including housework, because of fibromyalgia (0, no impairment; 10, maximum impairment); F14-20, questions 14-20 of FIQ, which are a series of visual analog scales for rating the various symptoms characteristic of FMS (0, no impairment; 70, maximum impairment);  ^e^Ellipses indicate not enough information to make a recommendation. CI, confidence interval.
